# Prevalence of Parental Violent Discipline Toward Children: Findings From A Portuguese Population

**DOI:** 10.1177/08862605241230552

**Published:** 2024-02-13

**Authors:** Armine Abrahamyan, Sara Soares, Sílvia Fraga, Henrique Barros

**Affiliations:** 1 EPIUnit—Instituto de Saúde Pública da Universidade do Porto, Portugal; 2 ITR-Laboratório para a Investigação Integrativa e Translacional em Saúde Populacional, Porto, Portugal

**Keywords:** corporal punishment, socioeconomic background, parent-child conflict tactics scale, child-reported

## Abstract

Despite recognizing the detrimental impact of parental violence on children’s mental and physical health throughout their lives, violence remains an all-too-real part of life for many children around the globe. However, data on the child-reported prevalence of experienced family violence are scarce and primarily based on parental reports. This study aimed to broaden the body of evidence and measure the lifetime prevalence of child-reported experience of violent disciplinary practices perpetrated by parents and to identify its associated sociodemographic and economic factors. We conducted a cross-sectional study using data from 5,281 Generation XXI participants recruited from 2005 to 2006 in Porto, Portugal. Parental disciplinary practices were reported by 7-year-old children using the Parent-Child Conflict Tactics Scale. Pearson’s Chi-squared test was used to compare differences in child-reported frequencies of violent disciplinary practices by sociodemographic variables. We observed statistically significant differences in rates of violent disciplinary practices according to the child’s and parent’s gender. Specifically, fathers exhibited a higher likelihood than mothers to engage in psychological aggression and corporal punishment, while mothers were more prone to engage in severe and very severe physical assault. When fathers were the perpetrators, boys were more inclined than girls to report all forms of violent disciplinary measures, and when mothers were the perpetrators, boys were particularly susceptible to severe and very severe physical assault compared to girls. In our study, children reported being frequently subjected to violent parental disciplinary practices, independently of family socioeconomic background. Children were more likely to experience psychological aggression and corporal punishment if they were born into high-income families, while severe and very severe physical assaults were more common among children whose parents had lower educational levels. National public awareness of the negative effects of violent disciplinary practices is urgently needed, promoting child-friendly and nonviolent approaches to discipline.

## Introduction

Violence against children is a problem in all countries and societies (Hillis et al., 2016). It involves different types of interpersonal violence that may occur at different stages in children’s development. Child maltreatment, which includes all types of abuse and neglect perpetrated by a parent, caregiver, or another person in a custodial role, results in actual or potential harm to the child’s health, survival, and development. Also, this type of violence mainly occurs at home, where children are meant to be protected and their rights guaranteed (UNICEF, 2010).

Child maltreatment is difficult to study, and existing estimates vary widely depending on the country and the method of research used. In Europe, physical abuse was much higher for boys than for girls (boys: 27.0%; girls: 12.0%). In North America, median rates were similar for boys and girls (boys: 24.3%; girls: 21.7%). Median prevalence rates of emotional abuse were nearly double for girls than boys in North America (girls: 28.4%; boys: 13.8%) and Europe (girls: 12.9%; boys: 6.2%). Regarding neglect, median rates were highest in Africa (girls: 41.8%, boys: 39.1%) and South America (girls: 54.8%, boys: 56.7%) (Moody et al., 2018). Violent discipline used at home by parents or caregivers is the most common form of violence experienced by children. Three-quarters of the world’s children aged two to four are regularly subjected to violent discipline by their parents or other caregivers (UNICEF, 2017), and around 6 in 10 children aged 2 to 14 are periodically punished physically (UNICEF, 2014).

### Parental Violent Discipline

Teaching children self-control, acceptable, and responsible behavior is integral to child rearing in all cultures. However, many parents or caregivers rely on violent methods, both physical and psychological, to punish unwanted behaviors and encourage desired ones. While children of all ages are at risk, experiencing violent discipline at a young age can be particularly harmful, given the increased potential for physical injuries and children’s inability to understand the motivation behind the act or to adopt coping strategies to alleviate their distress.

Compelling evidence links violent disciplinary practices, like corporal punishment, to a wide range of negative short-and long-term outcomes, including psychosocial functioning, physical health, mental health, and alterations in physiological biomarkers (Fraga et al., 2022).

Over the last decade, despite recognizing the pervasive nature and impact of parental violence against children, parents have continued using it to address and correct children’s misbehavior (Bassam et al., 2018; Gershoff, 2002; Gershoff & Grogan-Kaylor, 2016). Worldwide, around one billion caregivers admit the necessity of physical punishment as a form of discipline (UNICEF, 2017). As of 2022, only 64 countries have entirely prohibited corporal punishment of children in all settings, including in homes (Global Partnership to End Violence Against Children, 2022).

### The Intergenerational Transmission of Violence

Understanding the factors associated with parents’ violent discipline decision-making can clarify the cycle of adversities, including the transmission of violence through harmful parenting practices (Lotto et al., 2021). The intergenerational transmission of violence theory proposes that parents abused as children are likely to be abusive to their children (Curtis, 1963). One possible approach to account for this intergenerational transmission involves social learning and role modeling theory (Bandura, 1977), whereby children who grow up witnessing and experiencing violence are more likely to be either perpetrators or victims later in their relationships. Further, Bowlby’s attachment theory (Broberg, 2007) emphasizes that the parent-child attachment relationship is the prototype for later relationships. For children growing up in violent households, security expectations are shattered as their protectors become their attackers, and there is nowhere to turn for help. Children who have experienced maltreatment or attachment-related difficulties are more likely to express hostility and anger toward others in various ways.

### Parental Discipline and Its Associated Factors

The parental violent discipline is influenced by a variety of factors and conditions, including parent characteristics (e.g., parents’ own experience of child maltreatment, age and education level, cognitive ability, personality), child characteristics (e.g., age, sex), and sociodemographic conditions (e.g., household income, number of children in the household) (Creavey et al., 2018; Friedson, 2016; Yunus & Dahlan, 2013). Families of low socioeconomic status (SES) tend to have more conflict, use harsher and more punitive discipline, and have inconsistent parenting (Chen & Miller, 2013; Roubinov & Boyce, 2017). Further, children from families of low SES are more likely to witness and experience violence not only inside their homes but also outside.

### Context of Portugal

Published data on the prevalence of violent disciplinary practices is scarce in Portugal. Machado et al. (2007) reported that in northern Portugal, one in four parents had perpetrated at least one act of emotional or physical abuse toward a child during the previous year. Ribeiro et al. (2016) reviewed forensic medical reports of alleged child abuse cases and reported that over half of the suspected intra-familial, physical abuse of children was due to corporal punishment. Another study reported retrospective accounts of parents’ childhood violence experiences, with over 70% reporting physical abuse as children (Figueiredo et al., 2004). Existing estimates rely on retrospective data and registries based on forensic reports.

However, there are no published data on young children’s self-reported experiences of parental disciplinary practices in Portuguese families beyond official and caregivers’ reports. In Portugal, the legal frame prohibits all forms of violence against children, including corporal punishment, in all settings (Portuguese Penal Code, Art. 152nd) (Diário da República Eletrónico, 2007). Nevertheless, corporal punishment seems to remain a socially tolerated means of disciplining children in Portugal (Ribeiro et al., 2016).

### Current Study

Violent discipline is a violation of children’s right to protection from all forms of violence while in the care of their parents or other caregivers. Measuring violence is challenging, particularly in research involving children. Children may lack prospective to describe and rate their subjective experiences of violence; however, using appropriate instruments to assess these events would contribute to understand the magnitude of this problem behind the tip of the iceberg.

The present study aims to estimate the lifetime prevalence of children reported victimization by parental violent disciplinary practices, using a large sample of Portuguese 7-year-old children, and to describe associated sociodemographic and economic factors (e.g., parental age, education, employment status, household income, family structure, and the number of siblings in the home).

## Methods

### Study Design, Settings, and Participants

This study was conducted using data from Generation XXI, a prospective population-based birth cohort study set up in Porto, Portugal (Alves et al., 2012). Briefly, mothers who gave birth to liveborn children with a gestational age of over 23 weeks from 2005 to 2006 were recruited up to 72 hr after delivery from the five public maternities that covered the metropolitan area of Porto, Portugal. A total of 91.4% agreed to participate, yielding an initial cohort of 8,647 children. Cohort participants were invited to attend follow-up assessments at ages 4, 7, 10, and 13 years (86.3%, 79.6%, 73.9%, and 54.0% participation, respectively). Each follow-up included face-to-face interviews to collect information about the family (demographic and socioeconomic characteristics, family structure, and parental medical history) and the child (medical history, physical symptoms, and health-related behaviors). A comprehensive physical examination of the children was also conducted.

The study protocol was approved by the University of Porto Medical School/ S. João Hospital Ethics Committee and registered with the Portuguese data protection authority. A signed informed consent according to the principles of the Declaration of Helsinki was required from all participants and signed by the legal guardian. Parents were also asked specific permission to allow their children to respond to the Conflict Tactics Scale Parent-Child version without the parent being present.

This study is a cross-sectional study based on the 7-year follow-up assessment. We considered eligible for the present study all children with complete data on parental disciplinary practices (e.g., psychological aggression, corporal punishment, severe and very severe physical assault). The final sample comprised 5,281 participants (2,553 girls and 2,728 boys).

Children included in the present study were not different from the remaining cohort regarding sex distribution (48.3% girls vs. 50.0% girls). However, participants included in this study belonged to families with higher household income (24.6% vs. 15.5% with >2,000 €/month), lived with both parents (83.8% vs. 51.9%), had more than one sibling (12.5% vs. 11.1%) than those excluded (see Supplemental Table 1).

### Measures

#### Child-Reported Parental Violent Disciplinary Practices

The Parent-Child Conflict Tactics Scale (CTSPC) was designed to capture different forms of disciplinary tactics used by parents when handling conflicts in the parent-child relationship (Straus et al., 1998). There are two CTSPC forms with similar items: a 22-item self-report form for parents and a child-report form consisting of 23 picture cards (Straus et al., 1998). For the present study, data from the picture-based CTSPC was used. The CTSPC has been adapted and validated in Portuguese-speaking contexts (Reichenheim & Moraes, 2006). The CTSPC covers acts of nonviolent discipline (four items), psychological aggression (five items), corporal punishment (six items), severe physical assault (four items), and very severe physical assaults (three items) directed toward children (Straus et al., 1998).

Children were asked to provide information on disciplinary practices ever used by parents (separately for mother-child and father-child conflict)through a face-to-face interview conducted by a trained interviewer in a private setting. A familiar and safe environment was ensured by conducting the interviews in the same facilities and having the same interviewers administer the questionnaires as in the previous assessments. As each item was presented in pictures depicting the acts of maltreatment, the interviewer read a description such as “This girl’s father shook her when she did something wrong. When you do something wrong, does your father shake you?.” If the response was affirmative, the interviewer presented a second card that showed the response categories in stacked circles that the child could point at. Answers to the child-report form items were rated using a five-point Likert-type scale, ranging from never to always. At least one incidence of parental violent disciplinary practices, whether psychological or physical, ever committed against children was considered as exposure to lifetime violence in this study. For analysis, child-reported discipline practices used by parents were recoded *never* if the child did not report any act of parental violent discipline or *at least once* if a child reported that parents ever used violent disciplinary tactics. The prevalence of very severe physical assault as an extreme form of violence against children is low in our study population; thus, for the analysis, we merged severe and very severe physical assaults. The nonviolent discipline subscale was excluded from the study.

#### Sociodemographic Characteristics

Information on parental age, education, employment status, and monthly household income was provided by mothers when their children were 7 years of age. Parental age was defined as the age of the mother and father in completed years and was categorized as <30 years of age, 30 to 44 years of age, and ≥45 years of age. Education was considered to be the number of successfully completed years of formal schooling and classified according to the International Standard Classification of Education 2011 classes (ISCED, 2011). The low educational level corresponded to 9 years or less of formal schooling, intermediate education to 12 years of formal education, and high education corresponded to more than 12 years. Parental employment status was coded as employed if mothers or fathers had full-time, part-time, or less than a part-time job, unemployed if they did not have paid job at the time of the survey, and other if they were unpaid family workers, students, retired people (pensioners), house workers, etc.

The monthly household income included salaries and other sources of income, such as financial assistance, rent, monetary allowances, and alimony for all the household. A low monthly household income was defined as 1,000€ per month or less, intermediate if between 1,001€ and 2,000€, and high if higher than 2,000€. The lower class was defined as the situation where both parents receive at least the minimum national wage (485.00 € in 2012–2014) (PORDATA, 2019).

The child’s family structure was defined as living with both parents and living in lone parenthood or with others if the child lives only with the mother, the father, or with other family members. The number of siblings at home was categorized into no sibling(s), one sibling, and more than one sibling.

### Statistical Analysis

Sample characteristics were summarized descriptively using counts and percentages. A χ^2^ test of independence with a statistical significance level of *p* < .05 was performed to compare differences in the reported occurrences of parental disciplinary practices by sociodemographic and economic characteristics, by mothers and fathers, and by sex of children. All statistical analyses were performed in R version 4.2.2 (R Core Team, 2023).

## Results

The sample characteristics of the study population are presented in Table 1. Around 80.1% of fathers and 83.7% of mothers were 30 to 44 years old, 49.2% of fathers and 38.6% of mothers had less than 9 years of schooling, 10.5% of fathers and 18.2% of mothers were unemployed, and 27.7% of these families had a low household income.

**Table 1.** Sociodemographic Characteristics of the Study Population, *N* = 5,281.

Characteristics, *n* (%)	Total *N* (%)	Girls *n* (%)	Boys *n* (%)
Father-related			
Age (years)			
<30	121 (2.6)	63 (2.8)	58 (2.4)
30–44	3,734 (80.1)	1,769 (78.4)	1,965 (81.7)
>45	807 (17.3)	425 (18.8)	382 (15.9)
Missing	619		
Education (years)			
≤9	2,317 (49.2)	1,118 (49.0)	1,199 (49.3)
10–12	1,340 (28.5)	648 (28.4)	692 (28.5)
>12	1,053 (22.4)	514 (22.6)	539 (22.2)
Missing	571		
Employment			
Unemployed	499 (10.5)	228 (10.0)	271 (11.1)
Employed	4,186 (88.5)	2,032 (89.0)	2,154 (88.0)
Other^a^	47 (1.0)	23 (1.0)	24 (1.0)
Missing	549		
Mother-related			
Age (years)			
<30	517 (9.8)	256 (10.0)	261 (9.6)
30–44	4,421 (83.7)	2,127 (83.3)	2,294 (84.1)
>45	343 (6.5)	170 (6.7)	173 (6.3)
Missing	0		
Education (years)			
≤9	2,017 (38.6)	977 (38.8)	1,040 (38.5)
10–12	1,602 (30.7)	773 (30.7)	829 (30.7)
>12	1,603 (30.7)	771 (30.6)	832 (30.8)
Missing	59		
Employment			
Unemployed	950 (18.2)	463 (18.4)	487 (18.0)
Employed	4,050 (77.5)	1,946 (77.1)	2,104 (77.9)
Other^a^	225 (4.3)	114 (4.5)	111 (4.1)
Missing	56		
Family related			
Household income (€/month)			
≤1,000	1,426 (27.7)	702 (28.2)	724 (27.1)
1,001–2,000	2,463 (47.8)	1,171 (47.1)	1,292 (48.4)
>2,000	1,267 (24.6)	615 (24.7)	652 (24.4)
Missing	125		
Family structure			
Lone parenthood or other	856 (16.2)	415 (16.3)	441 (16.2)
Living with both parents	4,418 (83.8)	2,135 (83.7)	2,283 (83.8)
Missing	7		
Number of siblings at home			
No siblings	1,931 (37.0)	933 (37.0)	998 (37.1)
1 sibling	2,633 (50.5)	1,282 (50.8)	1,351 (50.2)
>1 sibling	650 (12.5)	307 (12.2)	343 (12.7)
Missing	67		

a Other employment status included unpaid family workers, students, retired people (pensioners), house workers, etc.

The most frequently child-reported disciplinary practices were psychological aggression (77.1% by fathers; 76.1% by mothers), followed by corporal punishment (74.2% by fathers; 74.8% by mothers), severe and very severe physical assaults (25.6% by fathers; 23.4% by mothers) (see Supplementary Table 2). The parental practice most frequently reported as psychological aggression was “Shouted, yelled, or screamed at the child” (65.1% by fathers; 64.5% by mothers), followed by “Threatened to spank or hit the child but did not do it” (46.9% by fathers; 44.6% by mothers) (see Supplementary Table 2). “Spanked with a bare hand” item from the corporal punishment subscale was most frequently reported to be used by both fathers and mothers (60.5% and 59.9%, respectively). The item “Beat the child up, over and over as hard as mother/father could” accounted for most of the severe and very severe physical assaults in children’s reports (20.1% by fathers; 18.4% by mothers) (see Supplementary Table 2).

Children were reported to be subjected to psychological aggression and corporal punishment more frequently than their less privileged counterparts if they were born into high-income families (see Tables 2 and 3).

**Table 2.** Sociodemographic Characteristics According to Children’s Report of Paternal Disciplinary Practices, *N* = 5,281.

Paternal Disciplinary Practices, *n* (%)	Psychological Aggression				Corporal Punishment				Severe and Very Severe Physical Assault			
	Never	At least Once	χ^2^	*p*	Never	At least Once	χ^2^	*p*	Never	At least Once	χ^2^	*p*
Paternal age (years)			8.16	.017			24.67	<.001			5.97	.050
fig<30	20 (16.5)	101 (83.5)			31 (25.6)	90 (74.4)			96 (79.3)	25 (20.7)		
30–44	764 (20.5)	2,970 (79.5)			833 (22.3)	2,901 (77.7)			2,720 (72.8)	1,014 (27.2)		
>45	198 (24.5)	609 (75.5)			246 (30.5)	561 (69.5)			615 (76.2)	192 (23.8)		
Paternal education (years)			18.29	<.001			5.52	.063			9.33	.009
≤9	522 (22.5)	1,795 (77.5)			563 (24.3)	1,754 (75.7)			1,658 (71.6)	659 (28.4)		
10–12	293 (21.9)	1,047 (78.1)			325 (24.3)	1,015 (75.7)			1,019 (76.0)	321 (24.0)		
>12	171 (16.2)	882 (83.8)			219 (20.8)	834 (79.2)			783 (74.4)	270 (25.6)		
Paternal employment status			2.60	.272			1.24	.537			4.30	.116
Unemployed	118 (23.6)	381 (76.4)			125 (25.1)	374 (74.9)			347 (69.5)	152 (30.5)		
Employed	862 (20.6)	3,324 (79.4)			974 (23.3)	3,212 (76.7)			3,092 (73.9)	1,094 (26.1)		
Other^a^	9 (19.1)	38 (80.9)			13 (27.7)	34 (72.3)			34 (72.3)	13 (27.7)		
Household income (€/month)			36.62	<.001			25.21	<.001			0.30	.684
≤1,000	387 (27.1)	1,039 (72.9)			428 (30.0)	998 (70.0)			1,054 (73.9)	372 (26.1)		
1,001–2,000	565 (22.9)	1,898 (77.1)			610 (24.8)	1,853 (75.2)			1,833 (74.4)	630 (25.6)		
>2,000	220 (17.4)	1,047 (82.6)			276 (21.8)	991 (78.2)			948 (74.8)	319 (25.2)		
Family structure			98.07	<.001			124.97	<.001			30.96	<.001
Lone parenthood or living with others	308 (36.0)	548 (64.0)			352 (41.1)	504 (58.9)			703 (82.1)	153 (17.9)		
Living with both parents	900 (20.4)	3,518 (79.6)			1,007 (22.8)	3,411 (77.2)			3,225 (73.0)	1,193 (27.0)		
Number of siblings at home			26.36	<.001			32.64	<.001			33.24	<.001
No siblings	511 (26.5)	1,420 (73.5)			583 (30.2)	1,348 (69.8)			1,517 (78.6)	414 (21.4)		
1 sibling	527 (20.0)	2,106 (80.0)			599 (22.7)	2,034 (77.3)			1,916 (72.8)	717 (27.2)		
>1 sibling	150 (23.1)	500 (76.9)			161 (24.8)	489 (75.2)			445 (68.5)	205 (31.5)		

a This variable included unpaid family workers, students, retired people (pensioners), house workers, etc.

**Table 3.** Sociodemographic Characteristics According to Children’s Report of Maternal Disciplinary Practices, *N* = 5,281.

Maternal Disciplinary Practices, *n* (%)	Psychological Aggression				Corporal Punishment				Severe and Very Severe Physical Assaults			
	Never	At least Once	χ^2^	*p*	Never	At least Once	χ^2^	*p*	Never	At least Once	χ^2^	*p*
Maternal age (years)			12.74	.002			10.70	.005			1.71	.426
<30	145 (28.0)	372 (72.0)			140 (27.1)	377 (72.9)			406 (78.5)	111 (21.5)		
30–44	1,016 (23.0)	3,405 (77.0)			1,083 (24.5)	3,338 (75.5)			3,370 (76.2)	1,051 (23.8)		
>45	101 (29.4)	242 (70.6)			110 (32.1)	233 (67.9)			267 (77.8)	76 (22.2)		
Maternal education (years)			2.21	.332			0.11	.944			11.49	.003
≤9	500 (24.8)	1,517 (75.2)			507 (25.1)	1,510 (74.9)			1,496 (74.2)	521 (25.8)		
10–12	377 (23.5)	1,225 (76.5)			406 (25.3)	1,196 (74.7)			1,237 (77.2)	365 (22.8)		
>12	364 (22.7)	1,239 (77.3)			398 (24.8)	1,205 (75.2)			1,264 (78.9)	339 (21.1)		
Maternal employment status			2.38	.305			2.28	.321			0.47	.710
Unemployed	226 (23.8)	724 (76.2)			255 (26.8)	695 (73.2)			733 (77.2)	217 (22.8)		
Employed	952 (23.5)	3,098 (76.5)			998 (24.6)	3,052 (75.4)			3,097 (76.5)	953 (23.5)		
Other^a^	63 (28.0)	162 (72.0)			60 (26.7)	165 (73.3)			169 (75.1)	56 (24.9)		
Household income (€/month)			6.81	.033			2.10	.350			7.67	.022
≤1,000	354 (24.8)	1,072 (75.2)			376 (26.4)	1,050 (73.6)			1,054 (73.9)	372 (26.1)		
1,001–2,000	605 (24.6)	1,858 (75.4)			613 (24.9)	1,850 (75.1)			1,905 (77.3)	558 (22.7)		
>2,000	267 (21.1)	1,000 (78.9)			304 (24.0)	963 (76.0)			987 (77.9)	280 (22.2)		
Family structure			1.92	.166			1.73	.188			1.14	.286
Lone parenthood or living with others	221 (25.8)	635 (74.2)			232 (27.1)	624 (72.9)			668 (78.0)	188 (22.0)		
Living with both parents	1,040 (23.5)	3,378 (76.5)			1,100 (24.9)	3,318 (75.1)			3,370 (76.3)	1,048 (23.7)		
Number of siblings at home			15.02	<.001			5.94	.051			28.11	<.001
No siblings	515 (26.7)	1,416 (73.3)			522 (27.0)	1,409 (73.0)			1,545 (80.0)	386 (20.0)		
1 sibling	572 (21.7)	2,061 (78.3)			633 (24.0)	2,000 (76.0)			1,986 (75.4)	647 (24.6)		
>1 sibling	155 (23.8)	495 (76.2)			155 (23.8)	495 (76.2)			458 (70.5)	192 (29.5)		

a This variable included unpaid family workers, students, retired people (pensioners), house workers, etc.

Severe and very severe physical assaults were more frequently observed among those children whose parents presented lower educational levels (fathers: 28.4%, mothers: 25.8%) or children of parents with low household income (fathers: 26.1%, mothers: 26.1%) (see Tables 2 and 3). Furthermore, children reported experiencing more acts of violent disciplinary practices if they were living with both parents and had one or more siblings at home (see Tables 2 and 3).

Table 4 compares the prevalence of violent disciplinary practices perpetrated by fathers and mothers. When considering only one parent, more fathers (38.7%) than mothers (10.9%) were reported to be perpetrators of psychological aggression (χ^2^ (1, *N* = 5,281) = 1,377.22, *p* < .001), and corporal punishment (χ^2^ (1, *N* = 5,281) = 1,469.85, *p* < .001). In contrast, more mothers (25.7%) than fathers (10.6%) practiced severe and very severe forms of physical assaults (χ^2^ (1, *N* = 5,281) = 2,016.32, *p* = .000) toward their children (see Table 4).

**Table 4.** Prevalence of Maternal and Paternal Violent Disciplinary Practices, *N* = 5,281.

Maternal Disciplinary Practices	Paternal Disciplinary Practices				
		Never	At least Once	χ^2^	*p*
Psychological aggression	Psychological aggression				
	Never	773 (61.3)	489 (38.7)	1,377.22	<.001
	At least once	437 (10.9)	3,582 (89.1)		
Corporal punishment	Corporal punishment				
	Never	873 (65.5)	460 (34.5)	1,469.85	<.001
	At least once	487 (12.3)	3,461 (87.7)		
Severe and very severe physical assaults	Severe and very severe physical assaults				
	Never	3,613 (89.4)	430 (10.6)	2,016.32	<.001
	At least once	318 (25.7)	920 (74.3)		

Table 5 shows the prevalence of parental violent disciplinary practices by sex of the child. The frequencies of all types of violent disciplinary practices perpetrated by fathers were higher for boys than girls. This gender difference was only observed again when comparing severe and very severe physical assaults used by mothers toward boys (χ^2^ (1, *N* = 5,281) = 16.23, *p* < .001) (see Table 5).

**Table 5.** Prevalence of Parental Violent Disciplinary Practices, by Sex of the Child, *N* = 5,281.

	Never		At least Once			
Parental violent disciplinary practices	Girls	Boys	Girls	Boys	χ^2^	*p*
Paternal disciplinary practices						
Psychological aggression	625 (51.7)	585 (48.3)	1,928 (47.4)	2,143 (52.6)	6.71	.009
Corporal punishment	699 (51.4)	661 (48.6)	1,854 (47.3)	2,067 (52.7)	6.68	.010
Severe and very severe physical assault	2,007 (51.1)	1,924 (48.9)	546 (40.4)	804 (59.6)	44.89	<.001
Maternal disciplinary practices						
Psychological aggression	595 (47.1)	667 (52.9)	1,958 (48.7)	2,061 (51.3)	0.89	.346
Corporal punishment	624 (46.8)	709 (53.2)	1,929 (48.9)	2,019 (51.1)	1.59	.207
Severe and very severe physical assault	2,017 (49.9)	2,026 (50.1)	536 (43.3)	702 (56.7)	16.23	<.001

## Discussion

Despite the recognition of the detrimental impact of parental violence on the mental and physical health and well-being of children throughout their lives, violence remains an all-too-real part of life for many children around the globe. In a context where all forms of violence against children are forbidden, and domestic violence is a public crime, we hypothesize that the frequency of violence perpetrated by parents is expected to be low. Using data from a large sample of Portuguese children of a population-based birth cohort, our findings contribute to the existing body of evidence by reporting that parental violent disciplinary practices are highly prevalent among Portuguese 7-year-old children. Our results revealed that psychological aggression is the most common disciplinary tactic applied toward children, followed by corporal punishment. These findings are similar to those reported previously (Chan, 2012; Cui et al., 2016; Fu et al., 2019). Corporal punishment encompasses a broad range of acts that parents may administer toward their children, from shaking and pinching the child to fatal physical assault. The operational definition of corporal punishment adopted for the present study included “the use of physical force with the intention of causing a child to experience pain, but not injury, for the purpose of correction or control of the child’s behavior” (Straus, 1994, p. 4). The Portuguese legislation establishes the incrimination of corporal punishment under articles 152nd and 152nd-A of the Penal Code that states: “Whoever repeatedly, or not, inflicts physical or psychological ill-treatment, including corporal punishment, deprivation of liberty and sexual offenses, is punished with one to 5 years of imprisonment.” (Diário da República Eletrónico, 2007). Over 60% of 7-year-old children in our sample reported to be “spanked with a bare hand” by parents suggesting that despite the entire prohibition of corporal punishment (Diário da República Eletrónico, 2007), it remains a highly prevalent tactic among Portuguese parents. One plausible explanation stems from Portuguese cultural norms, wherein corporal punishment often remains unrecognized as a form of violence against children. This practice finds a degree of social acceptance and is deeply ingrained in familial child-rearing traditions. This prevailing societal disposition toward considering parents’ use of violent discipline as a legitimate means of child guidance presents a significant hurdle in obtaining comprehensive reporting on such incidents. At present, there is a dearth of available published data that tracks the evolution of public perspectives concerning these practices over time.

Concerning severe and very severe physical assault, our results may seem to be high. However, these results should be interpreted with caution. The item “Beat her/him up, over and over as hard as mother/father could” emerged as the primary contributor to instances of severe and very severe physical assaults in children’s accounts. It is worth considering that this specific item within the questionnaires administered to children could have led to some misinterpretation, potentially inflating the prevalence rates. This misinterpretation could be attributed to children associating the wording with various forms of violence that may not necessarily align with being repeatedly beaten as severely as a parent could. Although the translation of the item was accurate, it is important to acknowledge that the nuanced meaning might not be universally consistent among all respondents.

We operationalized parental violent disciplinary practices as instances of “never” versus “once or more,” a classification that might be viewed as stringent with regard to chronicity. However, it is important to underscore that our adherence to a zero-tolerance toward violence, as mandated by national law, precludes the acceptance of any degree of tolerance for such incidents. The use of the term “violent discipline” serves to underscore the paramount legal and ethical obligations surrounding the rights of children (Diário da República Eletrónico, 2007).

Across studies, the lifetime prevalence of parental violent disciplinary practices varies considerably. Sun (2021) found that corporal punishment (89.47%) was the most prevalent form of child maltreatment, followed by physical assault (80.21%), psychological abuse (65.0%), and neglect (52.26%) among primary school children in Shanghai, China. The prevalence of mother-reported psychological aggression was 70.5%, followed by corporal punishment (51.4%) and physical maltreatment (9.8%) in the Brazilian sample (Gebara et al., 2017). Castillo et al. (2020) reported that 53.7% of mothers and 43.4% of fathers used corporal punishment to discipline child misbehavior. Gabriela Barajas-Gonzalez et al. (2018), in a sample of Mexican and Dominican American immigrant families with young children, showed that 41.4% had been physically punished and 91.8% experienced verbal punishment (i.e., “scold or yell when your child misbehaves”). The wide variance in the prevalence of parental violent disciplinary practices was explained by several methodological factors, including the gender of the respondent and perpetrator, the definition of child maltreatment, the method of data collection, the population from which the research sample is drawn, etc. (Moody et al., 2018).

Children from different socioeconomic backgrounds experience different parenting practices. Previous studies showed that socioeconomic circumstances shape parenting. Parents from low socioeconomic backgrounds are more likely to follow authoritarian styles, have a poor understanding of child development, display less warmth, and show disapproval toward their children (Machado et al., 2007). In our study, children frequently reported experiencing parental violent disciplinary practices regardless of parental socioeconomic background b. However, we found some differences according to parents’ socioeconomic backgrounds: children from low socioeconomic backgrounds were more likely to be victims of more severe and very severe forms of discipline, whereas children from high socioeconomic backgrounds reported more psychological aggression and corporal punishment. Children of more educated parents are more frequently subjected to psychological aggression, shouting, yelling, screaming at a child, and calling a child offensive names such as “dumb” and “lazy.” It is known that mothers with at least some college education were more likely to use a higher level of psychologically aggressive parenting than those with less than high school (Zhang & Anderson, 2010). Possibly, when children move from kindergarten to primary school, the parents’ concerns shift from the child’s physical well-being to the child’s future success (Li et al., 2013). Some parents may adopt a demanding style in parenting and place a high emphasis on children’s educational accomplishments. Notably, more educated parents may have high expectations for their children’s academic performance, as they believe that a desirable career and a “bright future” are the inevitable results of academic solid achievements (Hsieh et al., 2017).

Most studies suggested that both genders of the parent and the gender of the child are essential to take into account when it comes to discipline, although the results have been inconsistent (Cui et al., 2016; Endendijk et al., 2016). We found statistically significant differences in rates of violent disciplinary practices according to the child’s and parent’s gender. Specifically, when only one parent acted as a perpetrator, fathers were found to be more inclined than mothers to employ psychological aggression and physical punishment. In contrast, mothers were more likely to use severe and very severe physical assaults.

Boys were more likely than girls to report all types of violent disciplinary practices if perpetrators were fathers and severe and very severe physical assault if perpetrators were mothers. The biosocial theory of sex differences provides rationales for gender-differentiated parenting practices (Wood & Eagly, 2002). According to this theory, mothers and fathers use different parenting strategies with boys and girls depending on boys’ and girls’ divergent gender roles. On the other hand, boys are more likely than girls to react with aggression and negative behavior to parental control, whereas girls are more likely to comply (Endendijk et al., 2017). These gender differences in child behavior may result in parents’ gender-differentiated use of harsher discipline in response to a child’s noncompliant or aggressive behavior.

We found that children reported experiencing more acts of violent disciplinary practices if they had one or more siblings at home. Possibly, in a large household, negative sibling relationships might lead to conflicts among children, which in turn may call for harsher parental disciplinary tactics compared to a household with a single child (Hallers-Haalboom et al., 2016). Another possible explanation is that in large families, parents may have less time and energy to address and correct each child’s misbehavior, which may result in practicing violent discipline. Economic hardship faced by large families may aggravate this situation (Douki et al., 2013).

Also, it should be noted that when in the same household, the child is exposed to violent disciplinary practices perpetrated by both parents, it may increase the chronicity of violence and exacerbate the child’s vulnerability in the face of multiple exposures to domestic violence.

### Study Strengths and Limitations

Using data from a large sample in the scope of a population-based birth cohort strengthens this study. Most published evidence on parental violent disciplinary practices relies on data from clinical or criminal justice cases, which may characterize different profiles compared to the general population (Machado et al., 2007; Ribeiro et al., 2016). Furthermore, data from research settings is generally based on a single family member’s report, often a mother, thus subject to reporting bias (Cheng et al., 2018; Gebara et al., 2017). Children’s report on parental violent disciplinary tactics is a fundamental source of information beyond official and caregiver reports. To the best of our knowledge, this is one of the few studies, including children’s reports on parental disciplinary practices using the CTSPC.

In the Generation XXI birth cohort, from which our sample is drawn, less than 5% of recruited mothers were born abroad. The lack of ethnic variability at the recruitment time is explained by the small number of foreign citizens (329,898) living in Portugal in 2006 and by the fact that most migrants in Portugal resided in Lisbon metropolitan area (Instituto Nacional de Estatística, 2007). Our study is a population-based investigation where children were selected independently of their gender, racial-ethnic identity, socioeconomic background, religion, and culture.

Meanwhile, some limitations should be considered when interpreting our findings. We studied the phenomenon from the perspective of children. It should be noted that 7-year-old children might be constrained by their limited language abilities when reporting violence. However, for the present study, we employed CTSPC picture cards which had been adapted and validated in Portuguese-speaking contexts (Reichenheim & Moraes, 2006). The utilization of CTSPC picture cards helps overcome potential cultural and linguistic barriers that might affect children’s ability to communicate their experiences, as well as reduce potential biases that can arise from variations in how questions are worded or interpreted. The utilization of picture cards not only visually portrays diverse conflict situations and tactics but also closely resembles real-life situations, thereby allowing children to express their feelings, thoughts, and experiences more accurately (Sierau et al., 2018).

Our study discussed and presented data on parenting as reported by children; consequently, the study was unable to explore the impact of the parental relationship dynamics or the potential control exerted by one parent over the other parent’s parenting methods.

Finally, the attrition over time resulted in the inclusion of a more socioeconomically advantaged group of participants resulting in a high prevalence of psychological aggression and corporal punishment in our sample. It is reasonable to anticipate that if participants from less advantaged socioeconomic groups were retained, we would likely encounter instances of more severe and even very severe violent disciplinary practices. Similar socioeconomic patterning of loss to follow-up has been observed in other studies (Goldberg, 2001; Strandhagen et al., 2010). Howe et al. (Howe et al., 2013) suggested that the impact of attrition on estimates of inequalities might be minimal when there is no direct causal link between the study outcome (e.g., parental violent disciplinary practices) and participation in the study. In such situations, any pre-existing socioeconomic discrepancies that were initially present would likely become more pronounced and potentially accentuate the existing socioeconomic gaps that were present within our study cohort.

Our study delved into the critical issue of child-reported violence, considering a diverse range of socio-economic contexts within our sample. The accounts provided by children from various socio-economic backgrounds shed light on the multifaceted nature of violence experienced by young individuals. These diverse narratives not only highlight the commonalities in the types and patterns of violence faced by children but also underscore the influence of cultural nuances and societal structures on their experiences. However, it is important to acknowledge the limitations of generalizability. Cultural nuances, legal frameworks, and specific contextual factors can vary widely, potentially influencing the manifestations and reporting of child-reported violence. Therefore, while our findings provide a valuable foundation, it is crucial for future studies to confirm and adapt these insights within the specific contexts they aim to address.

## Conclusion

Despite compelling evidence that violent disciplinary practices are associated with an elevated risk of detrimental child outcomes (Gershoff & Grogan-Kaylor, 2016), parental violent disciplinary practices are highly prevalent among Portuguese 7-year-old children, independently of their family socioeconomic background. Further, parental disciplinary practices varied by gender of the parents and sex of the children. Public awareness campaigns to publicize the law regarding corporal punishment and targeted programs to educate parents about child-friendly and nonviolent approaches to discipline seem to be precursors to changes in parents’ beliefs and behaviors regarding violent disciplinary practices.

## Supplemental Material

sj-docx-1-jiv-10.1177_08862605241230552 – Supplemental material for Prevalence of Parental Violent Discipline Toward Children: Findings From A Portuguese Population Supplemental material, sj-docx-1-jiv-10.1177_08862605241230552 for Prevalence of Parental Violent Discipline Toward Children: Findings From A Portuguese Population by Armine Abrahamyan, Sara Soares, Sílvia Fraga and Henrique Barros in Journal of Interpersonal Violence
