# Supplementary material for: Prevalence of Parental Violent Discipline Toward Children: Findings From A Portuguese Population
Source: J Interpers Violence. 2024 Feb 13;39(9-10):1881–904. doi: 10.1177/08862605241230552 (PMC10993632; doi:10.1177/08862605241230552)
Supplement: sj-docx-1-jiv-10.1177_08862605241230552 – Supplemental material for Prevalence of Parental Violent Discipline Toward Children: Findings From A Portuguese Population [file sj-docx-1-jiv-10.1177_08862605241230552.docx]

**Supplementary Table 1**

*Comparative analysis of included and excluded participants, N=8647*

| **Characteristics, n (%)** | **Included**  **n=5281** | **Excluded**  **n=3366** | ***p*** |
| --- | --- | --- | --- |
| **Child-related** |  |  |  |
| Sex |  |  | .138 |
| Girls | 2553 (48.3) | 1683 (50.0) |  |
| Boys | 2728 (51.7) | 1683 (50.0) |  |
| **Mother-related** |  |  |  |
| Age (years) |  |  | <.001 |
| <30 | 517 (9.8) | 716 (21.3) |  |
| 30-44 | 4421 (83.7) | 2464 (73.3) |  |
| >45 | 343 (6.5) | 180 (5.4) |  |
| Missings | 0 | 6 |  |
| Education (years) |  |  | <.001 |
| ≤9 | 2017 (38.6) | 254 (48.6) |  |
| 10-12 | 1602 (30.7) | 150 (28.7) |  |
| >12 | 1603 (30.7) | 119 (22.8) |  |
| Missings | 59 | 2843 |  |
| Employment |  |  | .034 |
| Unemployed | 4050 (77.5) | 380 (72.5) |  |
| Employed | 950 (18.2) | 116 (22.1) |  |
| Other^a^ | 225 (4.3) | 28 (5.3) |  |
| Missings | 56 | 2842 |  |
| **Father-related** |  |  |  |
| Age (years) |  |  | <.001 |
| <30 | 121 (2.6) | 97 (5.7) |  |
| 30-44 | 3734 (80.1) | 1271 (74.9) |  |
| >45 | 807 (17.3) | 330 (19.4) |  |
| Missings | 619 | 1668 |  |
| Education (years) |  |  | .924 |
| ≤9 | 2317 (49.2) | 159 (49.1) |  |
| 10-12 | 1340 (28.5) | 95 (29.3) |  |
| >12 | 1053 (22.4) | 70 (21.6) |  |
| Missings | 571 | 3042 |  |
| Employment |  |  | <.001 |
| Unemployed | 4186 (88.5) | 276 (83.9) |  |
| Employed | 499 (10.5) | 43 (13.1) |  |
| Other^a^ | 47 (1.0) | 10 (3.0) |  |
| Missings | 549 | 3037 |  |
| **Family-related** |  |  |  |
| Household income (€/month) |  |  | <.001 |
| ≤ 1000 | 1426 (27.7) | 233 (43.1) |  |
| 1001-2000 | 2463 (47.8) | 244 (41.4) |  |
| >2000 | 1267 (24.6) | 84 (15.5) |  |
| Missings | 125 | 2805 |  |
| Family structure |  |  | <.001 |
| Lone parenthood or other | 856 (16.2) | 273 (48.1) |  |
| Living with both parents | 4418 (83.8) | 295 (51.9) |  |
| Missings | 7 | 2798 |  |
| Number of siblings at home |  |  | .001 |
| No siblings | 1931 (37.0) | 250 (44.7) |  |
| 1 sibling | 2633 (50.5) | 247 (44.2) |  |
| >1 sibling | 650 (12.5) | 62 (11.1) |  |
| Missings | 67 | 2807 |  |

^a^ Other employment status included unpaid family workers, students, retired people (pensioners), house workers, etc.

**Supplementary Table 2**

*Children reports of parental violent disciplinary practices, N=5281*

|  | **Fathers** | | **Mother** | |
| --- | --- | --- | --- | --- |
|  | **Never** | **At least**  **once** | **Never** | **At least**  **once** |
| **Psychological Aggression** | 1210 (22.9) | 4071 (77.1) | 1262 (23.9) | 4019 (76.1) |
| Shouted, yelled, or screamed at the child | 1841 (34.9) | 3440 (65.1) | 1877 (35.5) | 3404 (64.5) |
| Swore or cursed at the child | 4242 (80.3) | 1039 (19.7) | 4472 (84.7) | 809 (15.3) |
| Said you would send the child away or kick a child out of the house | 5225 (98.9) | 56 (1.1) | 5189 (98.3) | 92 (1.7) |
| Threatened to spank or hit the child but did not do it | 2802 (53.1) | 2479 (46.9) | 2926 (55.4) | 2355 (44.6) |
| Called the child stupid or lazy or some other name like that | 4851 (91.9) | 430 (8.1) | 4899 (92.8) | 382 (7.2) |
| **Corporal Punishment** | 1360 (25.8) | 3921 (74.2) | 1333 (25.2) | 3948 (74.8) |
| Shook the child | 5019 (95.0) | 262 (5.0) | 5017 (95.0) | 264 (5.0) |
| Hit the child on the bottom with some hard object | 4743 (89.8) | 538 (10.2) | 4587 (86.9) | 694 (13.1) |
| Spanked the child on the bottom with bare hand | 2085 (39.5) | 3196 (60.5) | 2120 (40.1) | 3161 (59.9) |
| Slapped the child on the hand, arm, or leg | 3657 (69.2) | 1624 (30.8) | 3675 (69.6) | 1606 (30.4) |
| Pinched the child | 4944 (93.6) | 337 (6.4) | 4953 (93.8) | 328 (6.2) |
| Slapped the child on the face or head, or ears | 3253 (61.6) | 2028 (38.4) | 3231 (61.2) | 2050 (38.8) |
| **Severe and Very Severe Physical Assault** | 3931 (74.4) | 1350 (25.6) | 4043 (76.6) | 1238 (23.4) |
| Hit the child with a fist or kick him/her hard | 5099 (96.6) | 182 (3.4) | 5177 (98.0) | 104 (2.0) |
| Beat the child up that is hit the child over and over as hard as you could | 4220 (79.9) | 1061 (20.1) | 4308 (81.6) | 973 (18.4) |
| Hit the child on some other part of the body besides the bottom with some hard object | 4982 (94.3) | 299 (5.7) | 4981 (94.3) | 300 (5.7) |
| Threw or knocked the child down | 5182 (98.1) | 99 (1.9) | 5220 (98.8) | 61 (1.2) |
| Grabbed the child around the neck and choked the child | 5182 (98.1) | 99 (1.9) | 5208 (98.6) | 73 (1.4) |
| Burned or scalded the child on purpose | 5262 (99.6) | 19 (0.4) | 5267 (99.7) | 14 (0.3) |
| Threatened the child with a knife or gun | 5275 (99.9) | 6 (0.1) | 5276 (99.9) | 5 (0.1) |
